# Biologic Agents—A Panacea for Inflammatory Arthritis or Not?

**DOI:** 10.1155/2009/420759

**Published:** 2009-12-08

**Authors:** J. Ninan, Malcolm D. Smith, M. Dugar, Karen O'Brien, Michael Ahern

**Affiliations:** ^1^Division of Rheumatology, Repatriation General Hospital, Adelaide 5042, Australia; ^2^Division of Rheumatology, Flinders Medical Centre, Adelaide 5042, Australia

## Abstract

*Aim.* To describe the retention rates for biological therapies in patients with rheumatoid arthritis (RA), psoriatic arthritis (PsA), and ankylosing spondylitis (AS) in a clinical setting. *Methods.* All patients managed in a dedicated biological therapy clinic in a teaching hospital in Australia were assessed for continuation on biological treatments and reasons for switching to an alternative biological agent or cessation of treatment. *Results.* There was a lower retention rate for RA patients on biological therapies compared to PsA and AS patients and the retention rate for RA patients was lower than that reported in RCTs. *Conclusions.* The retention rate on biological therapies for RA patients was lower in the clinic setting than what is reported in RCTs. The reasons for the lower retention rate in the clinical setting are discussed but no clear determinants for nonresponse to biological agents were identifiable. These agents have very limited steroid sparing effects.

## 1. Introduction

The treatment of inflammatory joint diseases (rheumatoid arthritis (RA), psoriatic arthritis (PsA), and ankylosing spondylitis (AS)) has been revolutionised with the introduction of biological therapies. 

Biologic Therapy refers to treatment that boosts or restores the ability of the immune system to fight inflammatory arthritis, cancer, and other diseases.

The major biologic approaches in clinical use include agents that interfere with cytokine function and those that inhibit the “second signal” required for T cell activation as well as agents that deplete B cells.

Randomised clinical trials (RCTs) have demonstrated a remarkable consistency in clinical responses to all biological agents (Infliximab, Etanercept, Adalimumab, Rituximab, Abatacept, and Tocilizumab) with all agents producing ACR20, ACR50, and ACR70 responses around 60%, 40%, and 20%, respectively, at 1 year followup for RA patients [1–6]. The duration of these RCTs have varied between 52 weeks and 4 years [1, 3].

The retention rate for RA patients on biological agents is also high, varying from 57 to 88% in RCTs although at least one study [7, 8] has suggested that the retention rate is much lower in a nontrial setting, falling to as low as 39% three years after commencement of a biological agent. 

Coprescription of a disease modifying agent (DMARD), which is usually Methotrexate, seems to increase the clinical efficacy of and retention rates for biological therapies in RA [9–11] and the retention rates appear to be higher in the PsA and AS patient groups compared to the RA patient group [7, 8, 12, 13]. 

It has been noted that there is a selection bias in the inclusion of patients in RCTs of biological treatments with many patients who are treated in the “real world” not meeting the inclusion criteria for RCTs [14, 15]. The clinical response to and the retention rates on biological treatments appear to be significantly lower in those patients who do not meet the inclusion criteria for RCTs compared to those of patients who do meet these inclusion criteria. It has also been suggested that the RA patients treated in the “real world” have less disease activity when commencing biological agents and that this may explain the lower efficacy in this patient group [8]. However, other studies report much higher retention and efficacy rates in RA patients receiving treatment with TNF blockers [12, 16, 17].

Most RA patients included in RCTs are taking oral corticosteroids and it is not clear how effective treatment with biological agents is in allowing a reduction in or cessation of oral corticosteroid treatment [18–20].

The cost of biological treatment is subsidised by the Australian Government so access to biological treatments for RA, PsA, and AS is not limited by individual financial constraints. However, there are restrictions in access to biological treatments imposed by the government with RA and PsA patients requiring to have a minimum of 20 tender and swollen joints (or four major joints including elbows, wrists, ankles, and knees) along with a CRP > 15 mg/L and/or an ESR > 25 mm/hour. RA and PsA patients must demonstrate a 50% reduction in tender and swollen joints and a 20% reduction in ESR or CRP levels at 3 months and continue to demonstrate such a response every 6 months in order to continue on biological treatment. AS patients must demonstrate a BASDAI (Bath Ankylosing Spondylitis Disease Activity Index) > 4.0 with a CRP > 10 mg/L and/or an ESR of > 25 mm/hour and demonstrate a 50% reduction in BASDAI and a 20% reduction in ESR or CRP levels at 3 months to continue on biological treatments. This response must be continued at assessments done every 6 months to continue on biological treatments. Biological treatments are given at fixed doses and treatment intervals as defined by RCTs and there is no provision for altering the dose or frequency of treatments in the event of loss of clinical efficacy. Therefore, if patients do not meet the pre-defined response criteria, they are recorded as a treatment failure (either primary or secondary) and must be switched to an alternative biological therapy. Primary treatment failed was defined as the patient not demonstrating efficacy with treatment at any time while secondary treatment failure was defined as an initial demonstration of response to treatment which was subsequently lost on continued treatment with a biological agent.

This paper presents the results of a retrospective study of clinical outcomes and retention rates for all patients (RA, PsA, and AS) commenced on biological agents since the commencement of a dedicated biological therapy clinic at a teaching hospital in 2002.

## 2. Methods

A dedicated clinic in a major teaching hospital in Adelaide, South Australia, was established in 2002 to treat initially RA and then PsA and AS patients with biological therapies. This clinic drains patients from the Southern region of Adelaide, a city of 1.2 million people, with an estimated drainage population of 400 000 people. All patients are assessed and followed up exclusively in this clinic. All patients from the clinic who had received biologic therapies as of June 30 2008 were included in the study. Data was collected from medical records and Medicare application forms by the investigators and entered into a computerised database. Data collection included joint counts, patient global response, modified Health Assessment Questionnaire (HAQ), laboratory (Rheumatoid Factor (RF), anticyclic citrullinated peptide (anti-CCP), C reactive protein (CRP) and erythrocyte sedimentation rate (ESR)) and radiological outcomes for RA patients; joint counts, patient global response, ESR and CRP for PsA patients and Bath Ankylosing Spondylitis Disease Activity Index (BASDAI), ESR and CRP for AS patients. As this was primarily a clinical audit an ethics approval was not sought.

Primary treatment failed was defined as the patient not demonstrating efficacy with treatment at any time while secondary treatment failure was defined as an initial demonstration of response to treatment which was subsequently lost on continued treatment with a biological agent.

### 2.1. Statistical Analysis

Statistical analysis was done using Chi Square tests, Kaplan Meier survival studies using EPI info software.

## 3. Results

One hundred and forty patients with RA, PsA, or AS were treated with biological agents as of 30 June 2008 (Table 1). In the RA group (93), first biological agent offered was Etanercept (52), Adalimumab (35), Infliximab (5), or Anakinra (1). Forty one patients (44.1%) ceased the first biological agent commenced, with 29 of the 41 (70.7%) ceasing because of lack of efficacy (8 primary efficacy failures, 21 secondary efficacy failures). Twelve (29.3%) of patients ceased treatment due to side effects including significant skin rashes including injection site reactions, exacerbation of heart failure, and serious infections. Thus the initial retention rate for the first biologic agent was 55.9%.

The median duration of treatment with the first biologic agent was 17 months: Adalimumab 16, Etanercept 17, Infliximab10, Anakinra 23. 

 In the RA group 47 (50.5%) were taking Methotrexate, Leflunamide (5), Hydroxychloroquine (3), and Salazopyrin (4) as cotherapy. Also of note was that 50.5% of the RA patients continued to require low dose prednislone (mean dose 7.9 mg ± 4.8 mg) at 9 months for disease control despite attempts to slowly withdraw them from corticosteroid treatment.

39 of the 41 patients who failed first biological went on to a second agent. 18 (46.2%) of these continued to show an ACR50 response. There were 5 primary failures and 7 secondary failures in this group. Nine patients developed side effects to the second biologic agent. Median duration of treatment with the second biologic agent was 16.5 months, 18 months (Adalimumab), 26(Etanercept), 7(Infliximab).

The retention rate with the second biologic was 47.4% in those who were taking an additional DMARD usually Methotrexate compared to 45% in those not on any additional DMARD (*P* = .74).

19 of the 21 patients who failed the second biologic agent went on to a third agent including Rituximab(6) and Abatacept(5) of these 14 continue to respond, there was 1 secondary failure and 2 primary failure and 2 developed side effects.

Within the RA patient group, we were unable to identify any determinants of treatment failure as the numbers were small to perform additional analyses. Males had a slightly better chance of responding to the first biological therapy than females 58.3% versus 56.5% (*P* = .22) chi square 4.3, but there was no difference in the age or disease duration between the responders and nonresponders to the first biological agent used.

Disease activity, including mean CRP levels, was similar between the responders and nonresponders while there was actually a higher level of Methotrexate cotherapy in the nonresponders group (57%) compared to the responders (52%) although this was probably by chance (*P* = .58).

### 3.1. Psoriatic Arthritis (27)

Twenty seven patients with Psoriatic arthritis were treated with biologic agents 13(Etanercept), 6(Adalimumab), 8(Infliximab)

**Figure 1:**
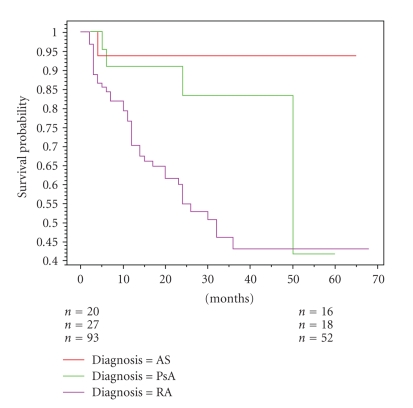
Kaplan-Meier survival curves for first biologic agent according to diagnosis *P*-value .0082.

The results were markedly different in this patient group compared to the RA group with only 9 patients ceasing the original biological agent: 1 primary treatment failure, 1 secondary treatment failure, 4 because of side effects (exacerbation of skin disease and infections), and 3 for other reasons including preference to a subcutaneous injection as opposed to infusion suggesting a response rate of 92.6% and retention rate of 66% to the first biologic agent (Figure 1).

These patients who discontinued the first agent were treated with a second agent. 7 of these patients continued to maintain good response. 1 failed and 1 developed side effects to the second agent. Both these patients are doing well on a third agent.

Only 12% of patients continued to take low dose corticosteroids.

### 3.2. Ankylosing Spondylitis (20)

In the AS patient group, all patients remained on biological therapies with 16 (80%) continuing on the original biological agent prescribed. Two of the patients switched to a different biological therapy because of side effects and two switched due to primary treatment failure.

## 4. Discussion

We describe in this paper our results of treatment with biological agents for RA, PsA, and AS from a single rheumatology unit in the setting of a dedicated biological therapy clinic. Our results are similar to those of Duclos et al. [7] with a higher retention rate on biological therapies in AS and PsA patients compared to RA patients. We have also showed that primary and secondary treatment failures result in around a third of patients failing to continue with treatment with the first and second biological therapy while retention rates were higher with the third biological treatment, although patient numbers in this group are low. There was however a tendency towards improvement in HAQ scores although this could not be analysed separately because of incomplete data.

These results are not as good as those seen in RCTs of biological therapies in RA. The reasons for the poorer response to biological therapies outside of RCTs are not clear but it has been suggested that selection bias, disease activity, patient choice or expense could all explain some of the differences seen [8]. It has also been suggested that the rigorous inclusion criteria for RCTs are not met by many of the patients who receive biological therapies in the clinic setting [14, 15] with the clinical response being suboptimal in those patients who did not meet RCT inclusion criteria [15]. The average age of the patients in this study was higher than that in RCTs as were the number of comorbidities but that is to be expected in the real world.

 In our study, we have not found any determinants which predicted nonresponders to biological therapies, including disease activity, coprescription of DMARDs, age, sex, or disease duration. It is possible that the stringent requirements that the Australian government has imposed for subsidisation of biological therapies may have had an effect on the biological therapy retention rate in our study, particularly as patients have to approach an ACR50 response to continue to receive subsidised treatment with biological therapies in Australia. However, another study of biological therapy in RA in Australia using only Etanercept has shown that 88% of patients remained on therapy with Etanercept at 12 months [21]. In addition, PsA patients have to meet similar response criteria to RA patients in order to receive subsidised biological treatment in Australia, yet we had a 92.6% response rate and a 66% retention rate to the first biological agent used in the PsA group in this study compared to 56% retention rate in RA (*P* = .008). Retention rates fall for subsequent TNF blockers from 56% to 46% and although there is some justification for switching agents one will eventually run out of agents with this trend. A recent report on outcomes of TNF blocker treatment in the South Swedish Arthritis Treatment Group Register has demonstrated similar results to our outcomes in this study with 26% of patients switching to a second TNF blocker with a 51% ACR20 and a 27% ACR50 response and a 13% switch to a third TNF blocker with a 35% ACR20 and a 18% ACR50 response [22]. This suggests that patients who fail to respond to the first TNF blocker are unlikely to do well on subsequent TNF blockers and consideration should be given to use an alternative biological agent, Abatacept, Rituximab, or Tocilizumab.

 It is possible that the inability to escalate dose or reduce the time between biological agent treatments in Australia could have resulted in the lower retention rate seen in this study. There is some evidence that treatment with Infliximab does demonstrate acquired drug resistance with a requirement for dose escalation over time [23], but this is unlikely to have affected our results significantly as the predominant biological therapies used in the RA group were Etanercept and Adalimumab. A recent trial has not demonstrated any benefit from dose escalation with Etanercept treatment [24].

We also demonstrated that over half the RA patients in this study were unable to come off corticosteroids, despite repeated efforts to slowly wean patients off corticosteroids while they were receiving treatment with a biological agent. Most of the RCTs do not measure withdrawal of corticosteroids as an endpoint in the studies so there is limited evidence that the use of biological agents in the treatment of RA has a corticosteroid sparing effect [18–20]. There are a number of potential reasons for why RA patients, more than PsA patients, are unable to reduce the use of corticosteroids, including the perception that biological therapies are not as effective in the RA group as they are in the PsA patient group.

Although this study has some limitations because of its retrospective observational design we believe it adds to our existing knowledge of biologic agent success and failures in the real world.

## 5. Summary

In summary, we have demonstrated in our clinic setting that the response to biologic therapies is greater in the PsA and AS group of patients than in RA patients and there is a lower retention rate on these agents in the RA group compared to what is seen in RCTs. The reasons for this lower retention rate are not readily apparent in this study.

## Figures and Tables

**Table 1:** Baseline demographic and clinical characteristics.

Diagnosis	RA (*n* = 93)	PsA (*n* = 27)	AS (*n* = 20)
Mean age (years ± sd)	62.35 (±12.4)	51.7 (±12.5)	48.8 (±9.2)
Sex (m : f)	24 : 69	15 : 12	15 : 5
Mean duration of illness (years ± sd)	14.7 (±10.3)	12.6 (±8.9)	12.6 (±11.3)
Tender and swollen joint count or BASDAI (AS patients)	25.9 (±7.4)	25.7 (±8.9)	7.8 (±1.59)
Mean CRP (mg/L)	43.5 (±39.1)	58.8 (±59.1)	47.1 (±44.0)
Mean ESR (mm/hr)	46.9 (±26.1)	36.8 (±18.6)	38.8 (±20.1)
% use of Corticosteroids	50.5	12	5
% use of Methotrexate	50.5	38.5	20
% chronic pain, Fibromyalgia	16.7%	15.8%	7.1%

sd: standard deviation, BASDAI: Bath Ankylosing Spondylitis Disease Activity Index, RA: Rheumatoid Arthritis, PsA: Psoriatic Arthritis, AS: Ankylosing Spondylitis, CRP: C Reactive Protein, ESR: Erythrocyte Sedimentation Rate.
